# Post-thoracotomy pain syndrome: seldom severe, often neuropathic, treated unspecific, and insufficient

**DOI:** 10.1097/PR9.0000000000000810

**Published:** 2020-03-04

**Authors:** Sven Arends, Andreas B. Böhmer, Marcel Poels, Marc Schieren, Aris Koryllos, Frank Wappler, Robin Joppich

**Affiliations:** a Department of Anaesthesiology and Intensive Care Medicine, University Hospital Essen and University of Duisburg-Essen, Germany; b Department of Anaesthesiology and Intensive Care Medicine, Cologne-Merheim Medical Centre, Witten/Herdecke University, Germany; c Department of Thoracic Surgery, Cologne-Merheim Medical Centre, Witten/Herdecke University, Germany

**Keywords:** Post-thoracotomy pain syndrome, Thoracotomy, Video-assisted thoracic surgery, Chronic postsurgical pain, Neuropathic pain

## Abstract

**Background:**

Post-thoracotomy pain syndrome (PTPS) is reported with a prevalence ranging between 33% and 91% in literature. However, the difference between open (TT) and video-assisted thoracic surgery (VATS) concerning the prevalence and neuropathic character of PTPS has not yet been systematically investigated. Furthermore, knowledge on analgesic treatment and its efficacy is limited.

**Methods:**

Structured telephone interviews were conducted with 488 patients 6 to 30 months after TT and VATS. In case of pain, patients received a structured questionnaire including the Leeds Assessment of Neuropathic Symptoms and Signs and Brief Pain Inventory.

**Results:**

Prevalence of PTPS was 28.6%. 13.2% of patients had a pain intensity Numeric Rating Scale >3, and 4.6% of patients had a pain intensity Numeric Rating Scale >5. In case of PTPS, 63% of patients suffered from neuropathic pain. Post-thoracotomy pain syndrome was more frequent after TT than after VATS (38.0% vs 29.3%, *P* < 0.05) and in patients younger than 65 years (42.3% vs 26.4%; *P* < 0.05). TT resulted more often in neuropathic pain (67.7% vs 43.9%; *P* < 0.05). Forty six percent of PTPS patients received analgesics: 30.3% nonopioids, 25.2% opioids, 10.9% anticonvulsants, and 1.7% antidepressants. Antineuropathic agents were used in 17.4% of patients with neuropathic pain. In 36.7% of patients, the reported reduction of pain was less than 30.0%.

**Conclusions:**

Post-thoracotomy pain syndrome is not as common as estimated. In most cases, pain intensity is moderate, but patients suffering from severe pain require special attention. They are often heavily disabled due to pain. Tissue-protecting surgery like VATS is beneficial for the prevention of PTPS. Analgesic medications are often underdosed, unspecific for neuropathic pain, and insufficient.

## 1. Introduction

Chronic pain is a common complication after thoracic surgery. The prevalence of post-thoracotomy pain syndrome (PTPS) ranges from 33% to 91%.^8,11,20,25^ Exact pathogenetic mechanisms for developing chronic pain after thoracotomy are unknown. Apart from intraoperative nerve damage and subsequent postoperative neuropathic pain, operation techniques, age, sex, pre-existing pain, genetic and psychosocial factors, severe postoperative pain, and analgesic management are suspected to have an impact on the development of PTPS.^16^ Considering the widespread use of minimal invasive surgical approaches, video-assisted thoracoscopic surgery provides an alternative to open thoracotomy for lung resection. However, its implications on the prevalence of PTPS have not yet been systematically investigated.

Our study aims to investigate the prevalence of PTPS in patients receiving open thoracotomy (TT) in comparison with video-assisted thoracic surgery (VATS). In case of PTPS, the presence of neuropathic pain as well as the use and efficacy of analgesic treatments was investigated.

## 2. Methods

The study was approved by the ethics committee of Witten/Herdecke University. The local hospital information system was used to identify all patients who underwent TT or VATS between 2009 and 2011. Chest wall resections were not included. After erasing duplicates, we applied our inclusion and exclusion criteria: Patients older than 18 years who received TT or VATS within the past 6 to 30 months and gave informed consent were included in the trial. We excluded patients who participated in other studies, had missing or invalid contact data, or were deceased.

All eligible patients were contacted by phone. The maximal number of attempts was 3. We performed a sample size calculation at the beginning of the study. After review of literature, prevalence of PTPS after TT was estimated to be 40%.^10,29^ As there were no data of PTPS after VATS available to us, based on our clinical experience, we expected that the prevalence would be lower compared with TT, due to decreased surgical trauma. For the purpose of our sample size calculation, we estimated the prevalence of PTPS for VATS to be <30%. Assuming this effect size in a one-tailed test for the difference between 2 independent mean values with alpha risk of 5% and beta risk of 20%, a sample size of 456 patients would be required. Thus, our aim was to contact 500 patients by telephone (250 TT and 250 VATS). The survey period was 6 to 30 months postoperatively.

All patients gave informed consent to participate in a short-structured telephone interview inquiring about current level of pain and discomfort. Chronic pain was defined as pain persisting past the normal time for healing.^3^ According to the IASP classification of chronic pain, 6 months was chosen as the point of division for acute and chronic pain.^18^ In case patients did not report any pain or discomfort, no further investigations ensued. In case of persisting pain or discomfort, written informed consent was obtained to include patients in a questionnaire-based survey containing the Brief Pain Inventory and the Leeds Assessment of Neuropathic Symptoms and Signs (LANSS). Brief Pain Inventory is a comprehensive and validated instrument for pain assessment.^22^ Pain was determined by Numeric Rating Scale (NRS 0–10). Pain-related disability was also assessed by the Brief Pain Inventory and categorised according to NRS scores. NRS scores are presented as mean (SD). After the initial telephone interview, all personal data were removed; the data set was collected and stored anonymously with a patient identification number.

### 2.1. Standard surgical and anaesthesiological procedure

The standard approach for TT was anterolateral thoracotomy. The incision was made on the level of the fourth or fifth intercostal space. The latissimus dorsi muscle was visualized and spared. The serratus anterior muscle was divided only when needed parallel to the muscle fiber direction, and the intercostal place was opened at the lower edge of the fourth or fifth rib. The vascular nerve bundle was carefully detached from the upper rib, and the intercostal nerve was resected at the level of the thoracotomy just laterally from the intervertebral foramen. A rib retractor was placed and gradually widened (maximal 7–10 cm). In case of VATS procedures without anatomical resection, three 1-cm ports (ventral and dorsal axillary line) were used. In case of VATS with anatomical resection (lobectomy and segmentectomy), two 1-cm ports and an axillary 3- to 4-cm incision in the fourth intercostal place were used. Rib retractors were not used in the VATS group. The opened intercostal space was closed using a continuous suture (thoracotomy and axillary incision in VATS group) sparing the intercostal nerve. VATS converted to TT was processed as TT in the statistical analysis.

Patients after TT received a paravertebral single-shot analgesia in landmark technique after closure of the operation wound by the anaesthesiologist combined with systemic analgesia based on the department's standard operating procedures for the management of acute pain after thoracic surgery. Patients after VATS received the same systemic analgesia. Regional anaesthesia was not used.

### 2.2. Analgesic treatment

Pain medications were categorised according to the following active agents: “nonopioid analgesics,” “opioid analgesics,” “anticonvulsive agents,” and “antidepressant agents.” Within the groups, the dosage was classified with respect to the recommended minimal and maximal dosage: “underdosed,” “low dosed,” “middle dosed,” “high dosed,” and “overdosed” (Table 1). For opioid analgesics, the categories “overdosed and underdosed” were not assigned, as they were titrated to effect.

**Table 1:**
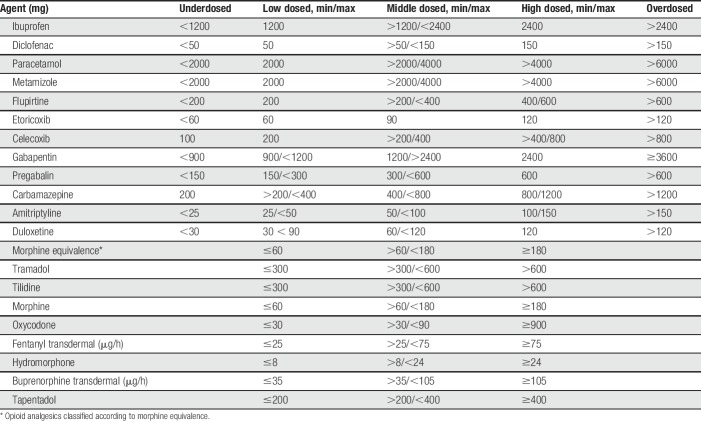
Dosage categories of oral analgesic agents (daily dosage).

### 2.3. Statistics

Professional statistical consulting services were provided by the university. Missing variables were completed if possible. Differences in categorical variables were calculated using the χ^2^ test. Continuous variables were calculated using the Mann–Whitney *U* test. For analysing predictors of PTPS, logistic regression analysis was performed. The occurrence of pain was considered a dependent variable, while age (<65 years/≥65 years), sex, surgical procedure (TT/VATS), presence of malignant primary disease, and postoperative period were considered independent variables. The significance threshold was α = 0.05 (*P* value ≤0.05).

## 3. Results

### 3.1. Enrolment

**Figure 1.**
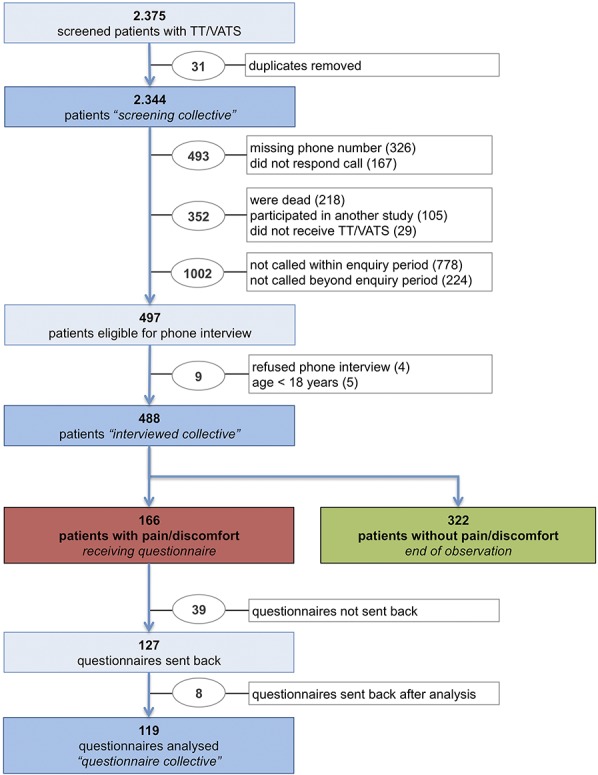
Flowchart of inclusion and patient collectives.

An overview of the enrolment process is outlined in Figure 1. Demographics of included patients are presented in Table 2. The cohorts of interviewed and screened patients were comparable with regard to sex, age, ratio of TT/VATS, and presence of cancer. The questionnaire response rate was 76.5%. Mean days since discharge were 460.0 (SD 187.0).

**Table 2:**
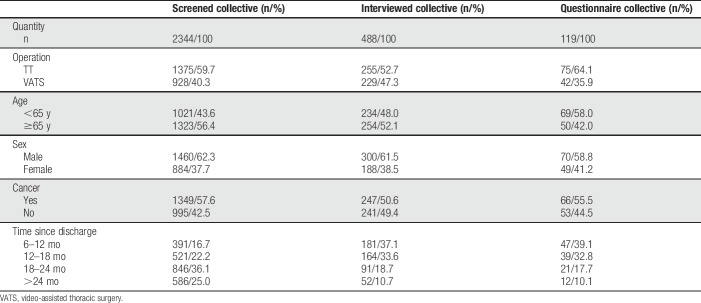
Patient characteristics.

### 3.2. Prevalence, pain intensity, and predictors of post-thoracotomy pain syndrome

The prevalence of PTPS with NRS ≥1 was 28.6%. 13.2% of patients experienced chronic pain with NRS >3. Pain levels of NRS >5 were reported by 4.6% of patients. Patients younger than 65 years suffered more frequently from PTPS than older patients (42.3% vs 26.4%; *P* < 0.05). After TT, PTPS was reported more often than after VATS (38.0% vs 29.3%; *P* < 0.05). There were no significant differences in rates of PTPS with regard to sex, presence of cancer, or time since the operation (Table 3). We performed a multivariate logistic regression analysis: TT and younger age were associated with a higher risk for development of PTPS. In females and cancer patients, however, these differences were not statistically significant (Table 4). In case of PTPS, pain intensity did not differ with respect to age, surgical technique, sex, presence of cancer, and postoperative period.

**Table 3:**
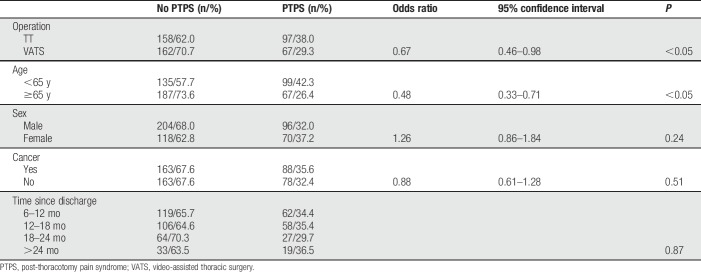
Prevalence of PTPS in subgroups (obtained from telephone interview).

**Table 4:**
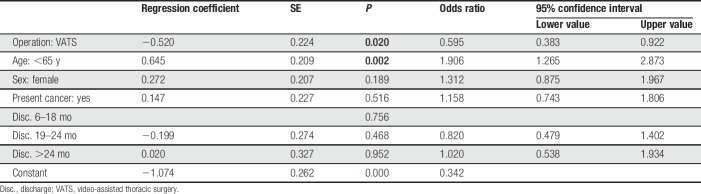
Logistic regression analysis.

### 3.3. Analgesic treatment

**Figure 2.**
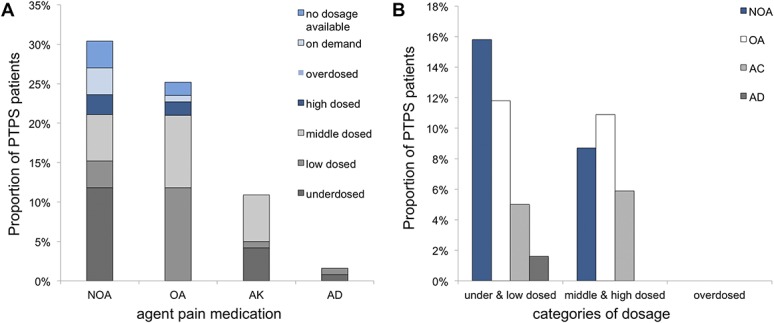
Groups and dosing of pain medication subscribed in case of PTPS. AC, anticonvulsant agents, AD, antidepressant agents; NOA, nonopioids, OA, opioids; PTPS, post-thoracotomy pain syndrome.

Pain medication was prescribed in 46.2% of PTPS patients. Patients with an average pain intensity of NRS >3 received analgesic treatment in 67.0%. Most patients received nonopioids and opioids (30.3%, respectively, 25.2% of questionnaire collective), 10.9% anticonvulsants, and 1.7% antidepressants. For details, see Table 5 and Figure 2.

**Table 5:**
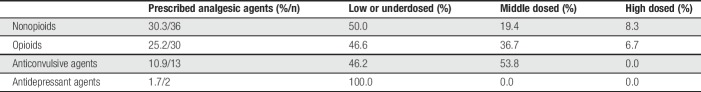
Prescribed analgesic agents and dosage categories.

**Figure 3.**
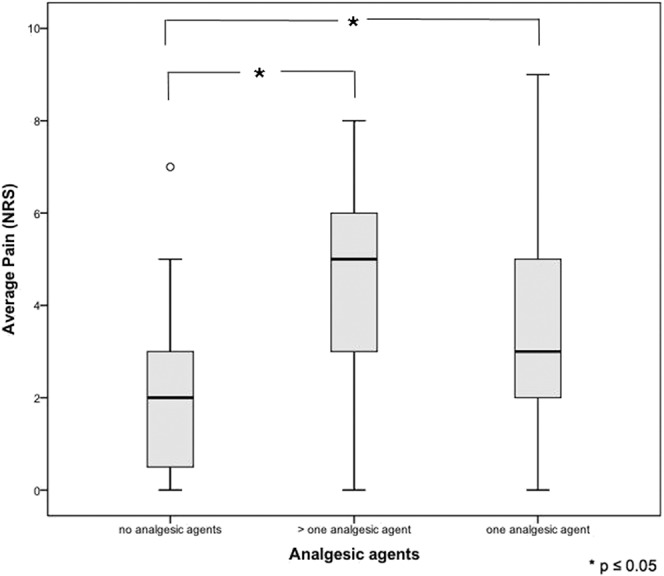
Analgesic combination therapy and pain intensity.

While 60.0% of patients were treated with analgesic monotherapy, 40.0% received more than one agent for pain control. Combination therapy consisted most frequently (68.2%) of nonopioids and opioids or of opioids and anticonvulsants (40.9%). The majority (70.9%) of patients receiving analgesic agents had no prescription for medication on demand. Average pain in patients with analgesic agents was significantly higher than in patients without analgesic agents (NRS presented as mean [SD]: patients with analgesic agents = 4.1 [2.1]; patients without analgesic agents = 2.1 [1.8]; *P* < 0.05). The more different analgesic agents were prescribed, the higher the mean pain (Fig. 3).

Information on the reduction of pain due to analgesic medication was provided by 49 patients. Mean pain reduction was 48.8% (27.6). In 36.7% of patients, the reported reduction of pain was less than 30.0%.

### 3.4. Neuropathic pain

**Figure 4.**
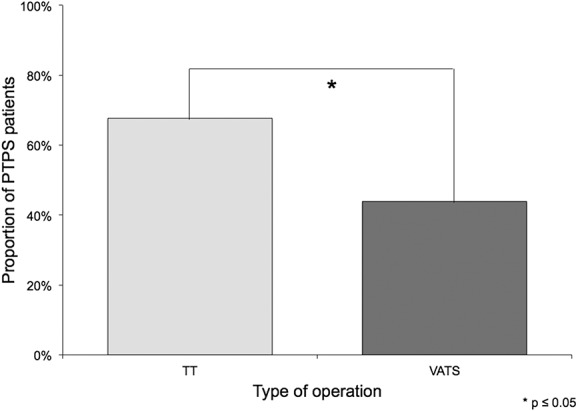
Probable neuropathic pain after TT and VATS. VATS, video-assisted thoracic surgery.

**Figure 5.**
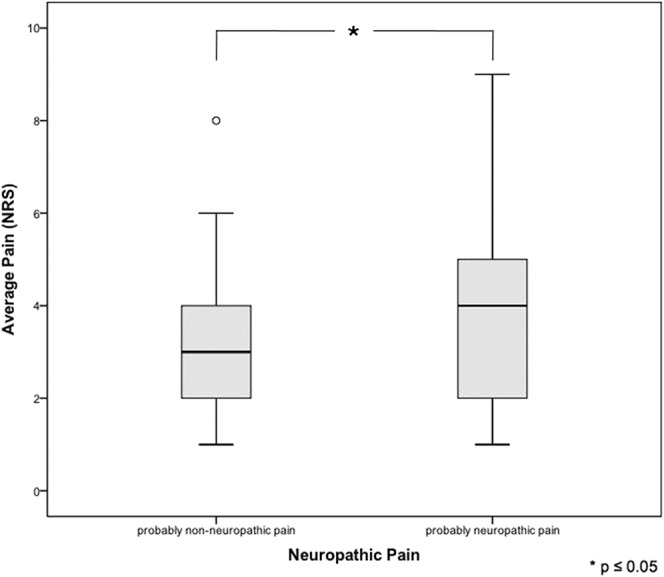
Pain intensity in patients with or without neuropathic pain.

**Figure 6.**
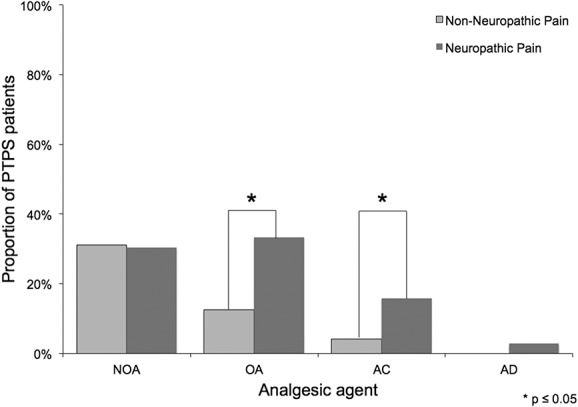
Prescribed analgesic agents in patients with and without neuropathic pain. AC, anticonvulsant agents; AD, antidepressant agents; NOA, nonopioids, OA, opioids.

According to LANSS, 63.0% of patients with PTPS suffered from neuropathic pain. In patients receiving TT with standard neurotomy, neuropathic pain was more frequent than in patients with VATS without neurotomy (67.6% vs 43.9%; *P* < 0.05; Fig. 4). Patients with malignant diseases complained more frequently of neuropathic pain (67.7% vs 48.1%; *P* < 0.05). In case of PTPS, pain intensity was significantly higher in patients with neuropathic pain than in patients without (*P* < 0.05 for average pain; Fig. 5). Post-thoracotomy pain syndrome patients suffering from neuropathic pain received analgesic agents in 52.4% of the cases (n = 33). Of those, 63.6% were prescribed nonopioids, 69.7% opioids, 33.3% anticonvulsant agents, and 6.1% antidepressant agents. Prescribed anticonvulsant agents were underdosed in 45.5% of patients. All prescribed antidepressant agents were underdosed or low dosed. Patients with neuropathic pain received more opioids and more anticonvulsant agents than patients without (Fig. 6). Antineuropathic agents (anticonvulsant or antidepressant agents) were used by 17.4% of patients with neuropathic pain (Table 6).

**Table 6:**
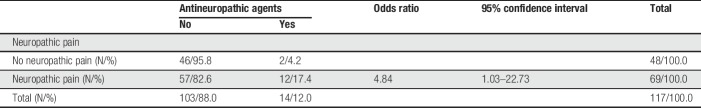
Antineuropathic agents in neuropathic pain states.

### 3.5. Pain-related disability

Mean pain-related disability of patients suffering from PTPS was between NRS = 2.14 and NRS = 4.08 depending on category of disability. Only a minority of PTPS patients (4.0%) had no impairment in any category. Patients with a low rating (NRS ≤3) were significantly less disabled due to pain than patients with an NRS rating >3 in all categories (*P* < 0.05; Table 7). Regarding age, surgical technique, sex, presence of cancer, and postoperative period, there were no differences in any category of pain-related disability.

**Table 7:**
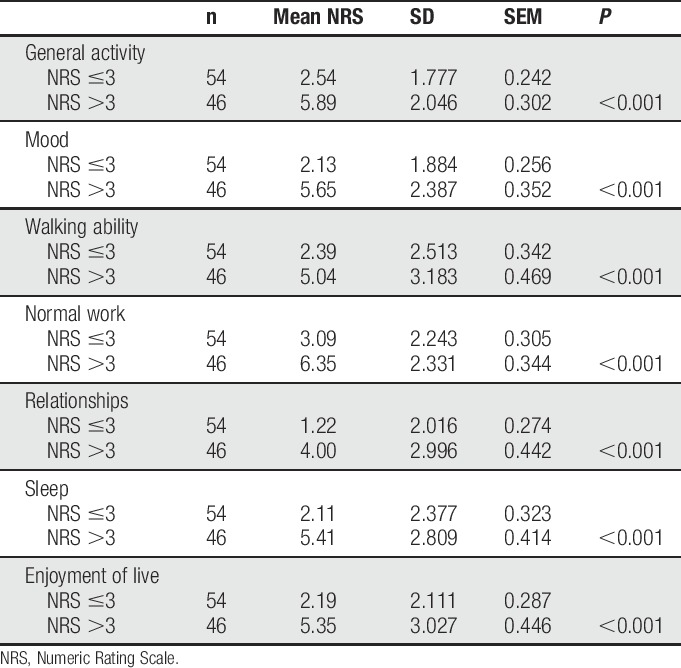
Pain-related disability in questionnaire collective.

## 4. Discussion

### 4.1. Prevalence and pain intensity

Our data showed that the prevalence of PTPS was not as common as estimated by previous studies. The prevalence of PTPS reported in the literature ranges from 33% to 91%.^8,29^ However, determination of the PTPS prevalence was not the primary purpose of these studies. Hence, different definitions of PTPS, survey periods and patient collectives (surgical technique, analgesic management, age, etc.) may have been potential confounding factors. A Danish nationwide questionnaire study determined a rate of 31.7%, as did a similar investigation in the United Kingdom (45.0%).^17,29^ Both studies had a response rate of 41.1% and 52.0%, respectively. It is tempting to speculate that affected patients may be more likely to respond to a survey, which could be a potential source for bias contributing to an overestimation of prevalence rates. We tried to reduce the impact of this potential confounder by performing telephone interviews. We assumed that patients without pain would be less likely to answer a questionnaire than patients suffering from pain. Subsequently, the use of a questionnaire might pose a risk for selection bias and might overestimate the prevalence of pain. We tried to minimise the risk for bias by contacting everyone by telephone and assess persistent pain or discomfort after the operation. Studies with higher prevalence rates are frequently associated with several methodical limitations, such as short postoperative survey periods, inclusion of elderly patients, routine performance of rib resections during thoracotomy, and postoperative analgesic management by morphine analgesia or by patient-controlled intravenous analgesia.^8,11,17,20,27^ At the time our study was performed, there was no consistent systematic classification of chronic postoperative pain available. We adhered to the IASP classification of chronic pain from 1994 and decided to include patients 6 months after surgery, as we wanted to ensure that pain-related disabilities of daily life had enough time to manifest. In the upcoming *ICD-11* classification, chronic postsurgical pain will be defined as pain persisting >3 months.^23^ This may limit the comparability to our findings. In addition, most previously reported prevalence rates result from surveys after posterolateral TT, which would be expected to lead to higher rates of PTPS than anterolateral TT, which was used in our investigation. In light of new developments in surgical and anaesthetic techniques, such as single-port VATS or regional anaesthetic techniques (eg, erector spinae block, pectoralis, and serratus plane blocks) might limit the external validity of our findings. By now, PTPS prevalence was estimated in synopsis of all data to be 30% to 40%.^10^

While PTPS is a frequent sequel of thoracic surgery, severe forms of PTPS occur less frequently. In a multicentre questionnaire-based trial, similar rates of moderate and severe PTPS were found: NRS >3 11.0% and NRS >5 4.0%.^29^ Another study assessed the severity of PTPS after posterolateral thoracotomy by telephone interviews.^21^ It found the overall prevalence of PTPS to be quite high (52%); however, patients reported moderate pain in 16% and severe postoperative pain in only 3% of cases. These results were confirmed by another study with an equally high prevalence of PTPS (61.0%) recorded 1 year after thoracotomy performed without epidural or paravertebral catheters, which reported an equally low rate of 5% for severe pain.^20^ Overall, the prevalence for severe PTPS seems to be low, although rates for moderate PTPS differ significantly.

### 4.2. Predictive factors

Minimally invasive VATS reduces tissue and nerve damage during surgery. Investigations comparing the prevalence of PTPS after TT vs VATS are rare. According to our data, TT leads more frequently to chronic post-thoracotomy pain than VATS. Once PTPS occurred, there was no difference in pain intensity with regard to the surgical technique used. One survey investigating chronic pain after TT and VATS found no differences 1 year after surgery; however, details on postoperative analgesia and response rates were not provided.^15^ In a registry analysis, TT and VATS were compared with a trend towards lower prevalence rates of PTPS after VATS, yet without statistical significance.^29^ In both trials, only patients with lung cancer were included and postoperative analgesia was provided by thoracic epidural anaesthesia for both TT and VATS. In other studies, the sample sizes were too small to draw valid conclusions.^6,12^ According to our data, age is a relevant predictive factor for developing PTPS with young patients (<65 years of age) being at a higher risk for chronic pain. This is in line with other investigations.^21,26,29^ Although the reason is unclear, limited inflammatory processes and higher pain tolerance in older patients may be possible explanations.

We could not detect any additional statistically significant predictive factor. Female sex is a known risk factor for developing chronic pain syndromes.^1^ The impact of sex on the incidence of PTPS, however, remains uncertain. Similar to previous results,^17^ we found a higher rate of PTPS in women. These differences were not statistically significant. An analysis of a larger cohort is required to further investigate the impact of patient sex.

Performing regional anaesthesia has shown beneficial effects regarding the development of chronic pain. Compared with intravenous PCA, patients with thoracic epidural analgesia developed PTPS less frequently.^27^ Paravertebral block seems to be effective as well.^13^ It is unclear, if the protective effect is based on effective postoperative pain management or on the regional anaesthesia technique itself.^9^ The use of regional anaesthesia in our study was affected by the surgical technique. Paravertebral single-shot regional anaesthesia was only used in patients with TT. Patients with VATS received systemic analgesia only. Thus, we cannot assess the impact of regional anaesthesia on the development of PTPS.

### 4.3. Analgesic treatment in case of PTPS

Knowledge on the analgesic management and its efficacy in case of PTPS is limited. Current investigations reported that between 33.0% and 53.0% of patients use analgesic medications.^8,17,26,29^ No further details are provided. 46.2% of our patients regularly used analgesics. Although 63.0% of patients suffered neuropathic pain with subsequently increased pain levels, antineuropathic medication was prescribed in only 17.4% of cases. By contrast, recommendations of national guidelines and current literature support systemic and topical antineuropathic treatment,^4,5^ which could improve pain therapy.

Dosing of analgesic agents was frequently inadequate because it was often underdosed or low dosed. Comparing average pain levels between patients being underdosed or low dosed and middle or high dosed, pain reduction in middle- or high-dosed patients tended to be better.

Specific and high-dosed antineuropathic medication could lead to better pain reduction. Pain management could be improved by adding short-acting analgesic agents on demand. However, 70.9% of all patients with analgesic medication had no additional medication on demand.

### 4.4. Neuropathic pain in PTPS

Considering that many patients suffering from PTPS report neuropathic symptoms^7^ and nerve damage may occur in thoracic surgery,^2^ neuropathic pain is a potential explanation for the development of PTPS. However, the exact role of neuropathic pain in PTPS is not entirely understood. Almost 2 thirds of our patients with PTPS suffered from neuropathic pain, according to questionnaire. LANSS is an established screening tool for neuropathic pain, which was also validated for the purpose of a questionnaire survey with high sensitivity and specificity.^28^ Patients reported neuropathic pain more frequently after TT than after VATS. Our high rate of neuropathic pain might be influenced by the extent of tissue and especially nerve damage. Another reason may be standard neurotomy performed in case of TT. The exact role of neurodestructive procedures like neurectomy or kryoneurolysis in the development of PTPS is unclear. Several studies failed to detect a beneficial effect of neurodestructive procedures on acute and chronic pain.^14,19^ Some patients even developed long-term neuralgia. This is in contrast to other studies showing less acute and chronic pain rates after neurodestructive procedures.^24^ Most studies used relatively short observation periods, which may not detect the late onset of long-term neuropathic pain.

Using other screening tools for assessing neuropathic pain does not significantly alter the prevalence of neuropathic pain.^8,26^ All rates are in accordance with our findings. However, the presence of neuropathic symptoms was associated with increased pain intensity and more impact on daily life activities, according to our data.^17^

## 5. Conclusion

PTPS is not as common as previously estimated. In most cases, pain intensity is moderate; nevertheless, patients suffering from severe pain have to receive special attention, as they are often heavily disabled by chronic pain. For the prevention of PTPS, tissue and nerve protective surgery seem beneficial. Accordingly, VATS should be performed if possible. Analgesic medication is often underdosed, unspecific for neuropathic pain, and insufficient.

## Disclosures

The authors have no conflicts of interest to declare.

Presented in part as a poster at the Congress of the European Federation of IASP Chapters (EFIC), Florence, Italy, October 2013.
